# An Electrochemical Chip to Monitor In Vitro Glycation of Proteins and Screening of Antiglycation Potential of Drugs

**DOI:** 10.3390/pharmaceutics12111011

**Published:** 2020-10-23

**Authors:** Zeeshan A. Khan, Seungkyung Park

**Affiliations:** School of Mechanical Engineering, Korea University of Technology and Education, Cheonan, Chunggnam 31253, Korea; acezeeshan@live.com

**Keywords:** diabetes, electrochemical chip, glycation, label-free, AGEs

## Abstract

Hyperglycemia and the production of advanced glycation end products (AGEs) are the primary factors for the development of chronic complications in diabetes. The level of protein glycation is proportional to the glucose concentration and represents mean glycemia. In this study, we present an electrochemical chip-based method for in vitro glycation monitoring and the efficacy evaluation of an antiglycation compound. An electrochemical chip consisting of five microchambers and embedded microelectrodes was designed for parallel measurements of capacitance signals from multiple solutions at different concentrations. The feasibility of glycation monitoring was then investigated by measuring the capacitance signal at 0.13 MHz with bovine serum albumin and gelatin samples in the presence of various glucose concentrations over 28 days. A significant change in the capacitance due to protein glycation was observed through measurements conducted within 30 s and 21 days of incubation. Finally, we demonstrated that the chip-based capacitance measurement can be utilized for the selection of an antiglycation compound by supplementing the protein solution and hyperglycemic concentration of glucose with an inhibitory concentration of the standard antiglycation agent aspirin. The lack of a significant change in the capacitance over 28 days proved that aspirin is capable of inhibiting protein glycation. Thus, a strong relationship exists between glycation and capacitance, suggesting the application of an electrochemical chip for evaluating glycation and novel antiglycation agents.

## 1. Introduction

**Figure 1:**
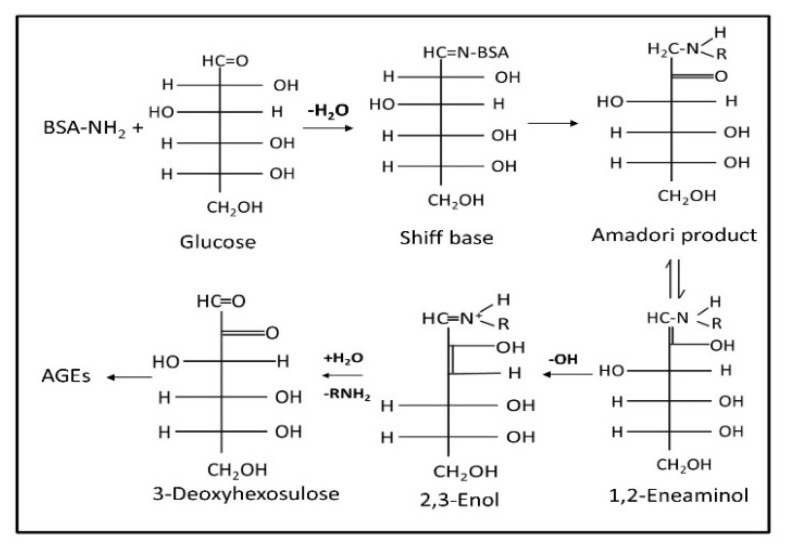
Advanced glycation end products by the Maillard reaction.

Diabetes is one of the most severe pathological conditions worldwide with 422 million afflicted patients, and this figure is rising dramatically, resulting in both health and economic challenges [1]. Hyperglycemia, a symptom that characterizes diabetes, leads to glycation of plasma proteins such as albumin, collagen, and hemoglobin. Atherosclerosis, aging, neurodegenerative diseases, and chronic renal failure are complex metabolic disorders associated with glycation induced by structural modifications in proteins (Figure 1) [2]. Glycated protein indicates an average amount of free glucose in the blood for an extended period, whereas the free glucose level may vary in the blood throughout the day [3].

A series of elaborate glycation events, known as the Maillard reaction, have been identified in many living organisms, including humans, leading to protein glycation. The Maillard reaction involves the formation of the Schiff base (aldimine adduct) by the reaction between the carbonyl group of reducing sugar and the side chain and N-terminal amino groups of a protein. Subsequently, the Schiff base undergoes tautomeric rearrangement to form ‘Amadori products’. These products are called the crosslinkers of the protein and glycosylating agents, generating advanced glycation end products (AGEs) (Figure 1), which in turn have several health implications [4]. Of the total serum albumin, 6–15% is glycated in normal conditions, and the amount of glycated protein increases with an increase in free glucose in the blood and in vitro, with 1% of glycated protein corresponding to an increase of 35 mg/dL glucose [5,6,7]. Drugs such as aspirin, metformin, and diclofenac were approved by the United States Food and Drug Administration as antiglycation drugs; nevertheless, these drugs are not potent enough to inhibit protein glycation in the case of chronic hyperglycemia [8]. Several other compounds such as ALT-711 and benfotamine are under investigation as potential antiglycation agents. Considering the increase in the diabetic population, there is a pressing need to develop antiglycation drugs with a higher efficacy; therefore, developing a device for the primary screening of novel antiglycation drugs is essential to facilitate drug discovery.

Generally, before in vivo studies, in vitro glycation is utilized for the primary screening of pro-glycation and antiglycation agents [9,10]. The extent of glycation in protein samples represents the potential of the drug; a decline in glycation indicates an antiglycation property, whereas an increase indicates glycation. High-performance liquid chromatography (HPLC), matrix-assisted laser desorption ionization time-of-flight (MALDI-TOF), and liquid chromatography–mass spectrometry (LC–MS) are conventionally used to study the level of glycation and antiglycation potential of a drug by measuring the extent of protein glycation [11,12]. A microfluidic capillary electrophoresis with mass spectrometry-based detection (CE-MS) was applied to measure hemoglobin glycation in whole blood lysate [13]. In another study, a microfluidic chip was utilized to enhance the rate of glycation-induced cross-linking of human scleral tissue as compared to conventional soaking [14]. The study showed that the microfluidic system can enhance the rate of in vitro glycation, but the fabricated chip was not capable of qualitative or quantitative determination of glycation. Utilizing the potential of the microfluidic chip, an impedometric sensor was fabricated for the detection of glycated hemoglobin (HbA1c) [15]. The microfluidic device has a pair of parallel facing gold electrodes to evaluate the impedance change and determine the concentration of HbA1c. The gold electrodes were modified with thiophene-3-boronic acid (T3BA) for the recognition of HbA1c. Similarly, another microfluidic system was developed to perform a chemiluminescence immunoassay-based detection of glycated HbA1c [16]. Anti-HbA1c antibody was used for specific recognition of HbA1c. As both microfluid chips have biorecongnition, they were used for evaluating of HbA1c glycation, and have never been utilized for the screening of antiglycation drugs. Additionally, the loss or denaturation of biorecongnition molecules might lead to false-negative results. Furthermore, quantitative screening of glycated proteins in various biological fluids and from eye lenses was successfully performed through LC–MS [17,18]. Although these are well-established and reliable tools, the requirement of expensive and heavy instrumentation, tedious sample preparation, use of toxic chemical reagents, and skilled personnel limits their wide acceptance. Efficient molecular assays including the enzyme-linked immunosorbent assay (ELISA) and other fluorometric and colorimetric assays such as nitroblue tetrazolium (NBT), thiobarbituric acid assays (TBA), and phenol sulphuric acid (PSA) for AGEs are sensitive and require a small sample volume [19]. NBT can be reduced by the high molecular mass of glycosylated proteins and contribute to the final signal [20]. TBA and PSA require the use of a corrosive reagent and a cumbersome hydrolysis process for sample preparation [21]. Furthermore, the use of engineered antibodies and fluorescent or colorimetric dyes, and a long sample processing time (dialysis before the assay), are other major issues in routine glycation analysis [19,22]. Moreover, most of the labeled reporter enzymes and dyes are sensitive to temperature; therefore, storage at an improper temperature causes degradation of the reporter molecules and loss of enzyme activity, leading to false-negative results or a high background noise. Recently, an approach based on the refractive index was presented for the label-free estimation of in vitro glycation of hemoglobin and albumin [23]. Despite its inexpensive and straightforward nature, the reflectometry-based approach was not useful for a higher concentration of proteins because of strong scattering and absorption. Autofluorescence of the glycated entities is also an interesting tool for analyzing glycation [24]; however, the majority of the AGEs are not fluorescent and thus cannot be detected. Moreover, the interference of non-AGE fluorophores is another challenge in using autofluorescence [25]. Even though cost-effective fluorometers are being developed, the cost of a fluorescence sensor is another hurdle, especially in developing and underdeveloped countries. Thus, the development of a miniaturized, label-free, simple, inexpensive, and reliable device for in vitro glycation is warranted.

**Figure 2:**
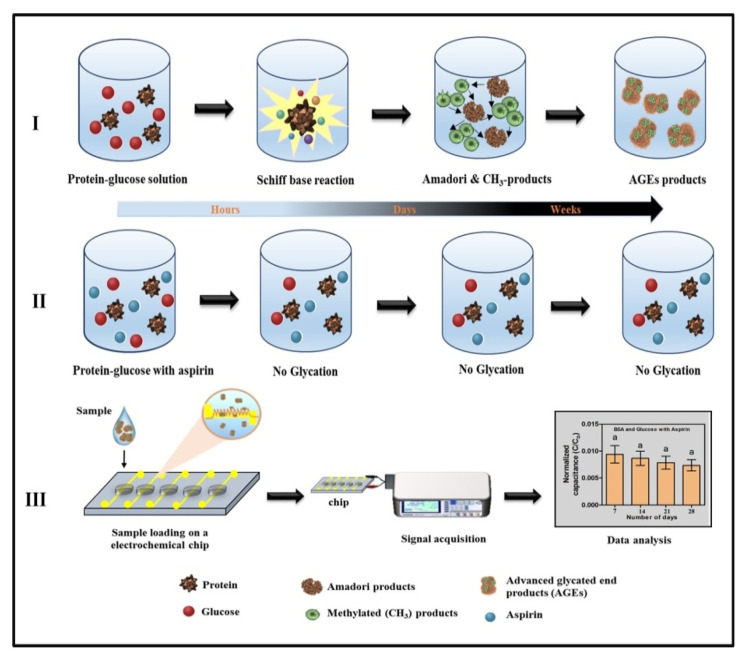
Schematic representation of in vitro glycation and electrochemical detection. (**I**) Glycation of protein in absence of antiglycation agent, (**II**) no glycation due to the presence of antiglycation agent aspirin and (**III**) detection of the glycation by electrochemical chip, change in normalized capacitance is observed in samples without aspirin and no change in capacitance was observed in samples with aspirin. Same letters indicate no significant differences between groups.

In this study, a label-free electrochemical detection method was developed for the determination of various protein concentrations in the solution in the presence or absence of glucose. Albumin is one of the major proteins that undergo glycation due to its high abundance in serum. Therefore, it is the most widely used protein to study the in vitro glycation. Bovine serum albumin (BSA) is a cost-efficient research strategy because it is inexpensive and possesses a high sequence and conformation homology with human serum albumin [26,27]. Moreover, BSA is considered as a model protein to investigate the effects of glycation, because of its abundance, and ease of purification [28,29,30]. Recently, several researchers have suggested that a novel agent can destroy AGE-derived protein cross-links. The first AGE breaker, *N*-phenacylthiazolium bromide, is unstable in vitro, and therefore, it was not clinically successful [31]. Following that, numerous herbal products have shown potent anti-glycation activities. For example, several polyphenols can inhibit the in vitro glycation process. Anti-glycation properties of various other plant metabolite flavonoids, such as genistein, kaempferol, quercitrin, and quercetin, have also been reported [32,33]. These compounds have capabilities to destroy the preformed glycated protein in vitro. This in vitro assessment is the primary step to understand the anti-glycation potential and mostly, NBT, ELISA, LC-MS-MS, HPLC, MALDI-TOF, etc. are utilized to study the in vitro glycation, which is either expensive or toxic. In this study, we attempt to simplify the in vitro screening of potent anti-glycation drugs. Research has shown that gelatin is a reliable surrogate for studying in vitro glycation as it is cheap, amenable to glycation, and correlates well with the defining features of protein glycation [34]. Therefore, glycation of BSA and gelatin at the hyperglycemic level with and without aspirin was studied over 28 days (Figure 2). A simple chip-scale device with embedded microelectrodes and five microchambers, each requiring only 100 µL of the sample, was designed and tested for the miniaturization purpose. Cumulatively, our proposed approach for the detection of AGEs and selection of an antiglycation drug is label-free, miniaturized, direct (no-sample preparation step), and chemical-free, with results obtained in less than 30 s of sample loading.

## 2. Methods and Materials

### 2.1. List of Reagents

Polydimethylsiloxane (PDMS), BSA, glucose, aspirin, and sodium azide were obtained from Sigma Aldrich, St. Louis, MO, USA. Positive photoresist (S1805) and negative photoresist (SU8) were obtained from Microposit, Philadelphia, PA, USA, and Microchem, Westborough, MA, USA, respectively. Gelatin was purchased from Duksan Chemicals, Daejeon, Korea.

### 2.2. Fabrication of the Electrochemical Chip

**Figure 3:**
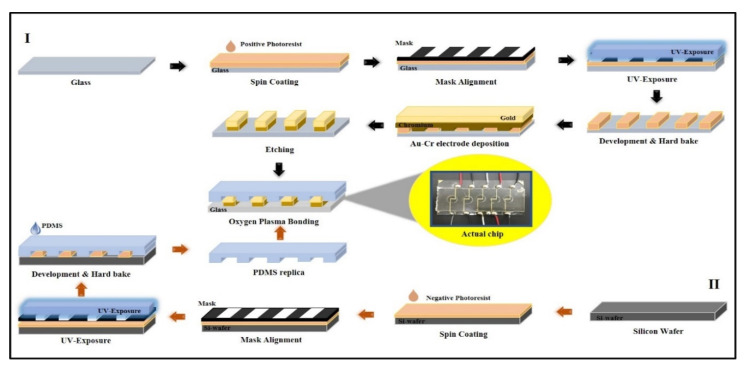
Sketches (not to scale) of the photolithographic process for the electrochemical chip fabrication. (**I**) Fabrication of gold-chromium electrodes on the glass surface and (**II**) fabrication of polydimethylsiloxane (PDMS) followed by plasma bonding with glass imprinted with electrodes.

Gold electrodes were deposited on a glass slide using conventional photolithography and the lift-off process. Briefly, the interdigitated electrode pattern with 100 µm spacing was designed with computer-aided design (CAD) software (AutoCAD v2018, Autodesk, San Rafael, CA, USA), and the mask was printed with a high-resolution printer on a transparency film. Positive photoresist (S1805) was spin-coated on the glass slide and exposed to UV light in a mask aligner (Midas, MDA-400S, Daejeon, Korea) to get the designed pattern. Using a sputtering machine (Q300T D, Quorum, East Sussex, UK), 15 nm chromium and 30 nm gold was subsequently deposited on the glass slide and lifted off to get the final electrode patterns. The electrochemical chip was fabricated using a standard soft lithography process published elsewhere [35]. Negative photoresist (SU-8) was used to achieve the designed pattern on the silicon wafer. PDMS was poured on the silicon wafer at a ratio of 10:1. The patterned PDMS was then gently peeled off from the wafer. The chamber on the chip was fabricated by punching holes into a PDMS slab with a 6 mm biopunch (Biopunch^®^, outer diameter 6.47 mm, Healthlink, Jacksonville, FL, USA). The fabricated electrode glass and PDMS chamber were aligned and bonded together by using an oxygen plasma of 30 W (Vita Plasma, Femtoscience, Gyeonggi, Korea) for 30 s. The chip was then heated at 60 ℃ for 2 h to achieve permanent bonding. The entire schematic of the completed electrochemical chip fabrication is presented in Figure 3.

### 2.3. Glycation of Proteins and Evaluation of Antiglycation Compound

The glycated BSA/gelatin solution embodying a high level of Amadori product was prepared following the published method by Cohen and Hud [36]. Three groups of BSA and gelatin were studied. Solutions of various concentrations of BSA, gelatin, and glucose were prepared in 20 mM phosphate buffer saline (PBS), pH 7.4 followed by filter sterilization (filter of size: 0.22 µm; CHMLAB, Terrassa, Barcelona, Spain). The first group comprised six concentrations of BSA (0, 1, 2.5, 5, 10, 20, and 40 g/L) and gelatin (0, 0.25, 0.5, 1, 2, 4, and 8 *w*/*v*). In the second group, 40 g/L of BSA or 2% gelatin was incubated with six concentrations of glucose (0, 150, 300, 450, 600, and 750 mg/dL) for 28 days on a shaker (Daihan Scientific, Wonju, Korea) at 37 °C under sterile and dark conditions. The samples were collected every 7 days for up to 28 days. For the evaluation of the antiglycation compound by using the electrochemical chip, a fixed concentration of 40 g/L BSA or 2% gelatin was incubated with the hyperglycemic concentration of glucose (300 mg/dL) and inhibitory concentration of aspirin (40 mmol/L) [9,26,34,37,38,39]. To inhibit the growth of any microorganism, 1 mmol sodium azide was supplemented in all the solutions. Intermittent sampling was performed to check for pH change and microbial contamination.

### 2.4. Electrical Signal Measurements and Data Analysis

The capacitance of the samples was measured using an LCR meter (E4890AL, Keysight Technologies, Santa Rosa, CA, USA), controlled in real-time by the LabVIEW 2017 program (National Instruments, Austin, TX, USA). After the sample was loaded, the fluidic chamber was sealed with a tape to prevent sample evaporation. The electrical signals of all the samples were measured in a stable incubator with a temperature of 37 °C. With an interval of 30 s, the sample was measured at 1 mV voltage and 1 kHz frequency. For all experiments, the capacitance values obtained from the measurement were normalized with the capacitance values of blank samples (without protein/glucose or with the zero-day samples in case of 28 days glycation studies). All the experiments were performed in triplicate, and three aliquots were taken from each sample to measure the capacitance.

## 3. Results and Discussion

### 3.1. Analysis of Glycation by Capacitance Measurements 

**Figure 4:**
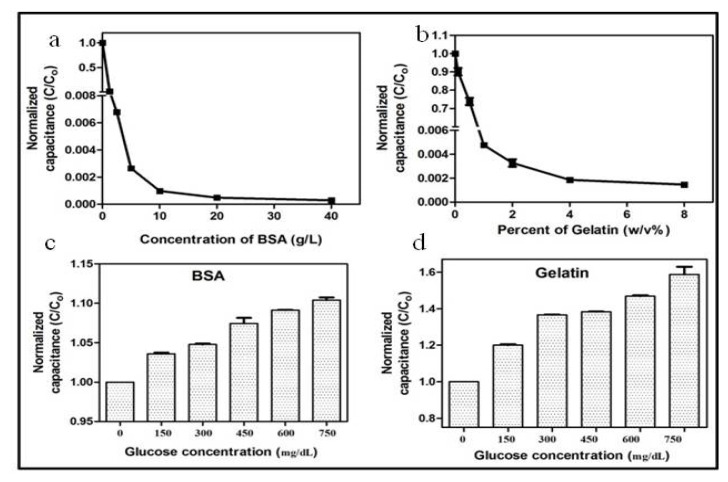
Normalized capacitance values of PBS solutions of (**a**) BSA (0, 1, 2.5, 5, 10, 20, and 40 g/L), (**b**) gelatin (0, 0.25, 0.5, 1, 2, 4, and 8% *w*/*v*), (**c**) 40 g/L BSA with different concentrations of glucose (0, 150, 300, 450, 600, and 750 mg/dL), and (**d**) 2% gelatin with different concentrations of glucose (0, 150, 300, 450, 600, and 750 mg/dL). The study was performed after 28 days of incubation. Error bars represent the standard deviation.

Figure 4 shows the experimental results of the normalized capacitance measurements of the different concentrations of BSA and gelatin, and solutions of 40 g/L BSA and 2% gelatin with different concentrations of glucose. The sample (100 µL) was pipetted in the microchambers, several frequencies ranging from 20 Hz to 0.3 MHz were tested, and optimum results were obtained at 0.13 MHz. The results for the entire range of frequency were generated within 30 s, whereas 10 s was required for the assessment of a single frequency. The normalized capacitance value increased with the change in the concentration of protein and glucose. The underlying principle behind the change in capacitance was speculated as being due to variation in the effective charges of the proteins and glucose, and the optimum value was observed at the specific frequency of 0.13 MHz. The increase in concentration adds more charged or polar molecules in the solution. Thus, the increase in capacitance was because of the change in the total surface charges on the electrode surface [40]. The extent of glycation was also confirmed by the NBT assay, which shows that a change in absorbance is directly proportional to the extent of glycation (Supplementary materials: Figure S1). In 1983, the NBT reduction assay for evaluating glycation of protein was first introduced by Johnson et al. [41]. The NBT is reduced by the ketoamine form of glycated protein, which causes a change in optical density at 525–530 nm [9,42]. Previously, the NBT assay was used to evaluate the level of glycation in albumin in the diabetic patients. The NBT method was correlated with the measurement of glycated albumin using boronate affinity chromatography with correlation coefficient (r) of 0.942 (*p* < 0.001) was observed [43]. In 2012, NBT analysis was utilized to detect the amount of glycated antibody [44]. Additionally, Wani et al. applied the NBT assay to analyze Amadori products (part of glycation process, Figure 1) in DNA [45]. Additionally, the AGE formation in the BSA samples was performed with the liquid chromatography mass spectroscopic analysis (LCMS) (Figure S2). N^ε^-carboxymethyl-lysine (CML), a well-known standard of AGEs, was used as a standard to study the formation of AGEs. The CML showed a peak at 279.1 *m*/*z*. A similar peak at 279.1 *m*/*z* was visible in the chromatogram of the 28 days incubated sample of BSA and glucose, whereas no peak was observed in the zero-day sample. These results confirm the glycation of BSA after 28 days. 

The current gold electrodes aim to detect the change in capacitance generated by the in vitro glycation of proteins. After a few hours of incubation of BSA or gelatin with glucose, the carbonyl group of a reducing sugar reacts with the free amino group of amino acids and generates a Schiff base. The Schiff base undergoes rearrangement to form the Amadori products by oxidation and glycation. The primary AGEs, CML, along with intermediates such as methylglyoxal (MG) and glyoxal (GO), are produced. The thickness of the electrical double layer increases with an increase in the size of the counterion, resulting in a decrease in capacitance [46]. Owing to the smaller size of free proteins and glucose in solution, a thinner double layer is formed, which is confirmed by higher normalized capacitance [40]. Amadori products and AGEs are generated with glycation, and bigger chemical species are formed due to their crosslinking. These species gradually increase the thickness of the electric double layer, thus decreasing the capacitance. The increase in the thickness of the electrical double layer might have been supplemented by the crosslinking of AGE, MG, and GO [47].

**Figure 5:**
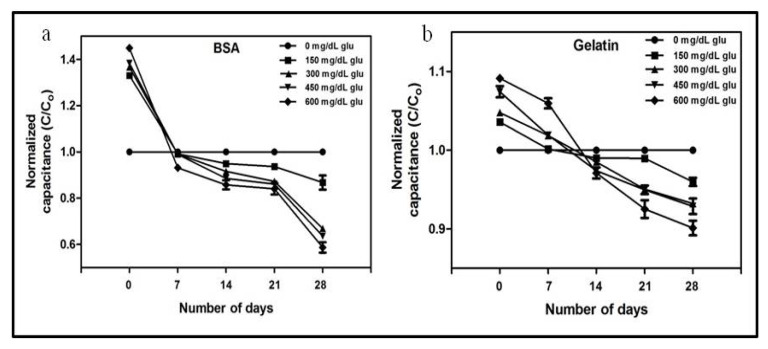
Experimental values of normalized capacitance of (**a**) 40 g/L BSA with various concentrations of glucose (0, 150, 300, 450, 600, and 750 mg/dL), and (**b**) 2% gelatin with various concentration of glucose (0, 150, 300, 450, 600, and 750 mg/dL). Incubation time is 28 days for both the samples. Error bars represent the standard deviation.

Figure 5 represents the change in the normalized capacitance of the BSA and gelatin with different concentrations of glucose over 28 days. Compared with the samples incubated without the glucose, the presence of glucose in samples leads to a considerable decrease in capacitance after 28 days of incubation. However, the PBS solution of protein without glucose remains constant throughout the 28 days of the experiment. Initially, the normalized capacitance of the protein solutions with glucose was higher than that of the protein solutions without glucose. With incubation, the capacitance of the PBS solutions of BSA and gelatin with and without glucose becomes approximately equal in 7–14 days, except for the gelatin solution with 150 mg/dL glucose, probably because 150 mg/mL of glucose was not enough to cause substantial changes in the 2% gelatin solution. A significant decrease in the capacitance was observed after 21 days of incubation, which became more prominent after 28 days. Moreover, the overall decrease in the capacitance of both BSA and gelatin was more prominent with the samples containing a higher concentration of glucose. A similar type of reaction occurs in the biological system—hyperglycemia induces the glycation of circulatory proteins proportional to the blood glucose level [48]. Thus, the decreasing normalized capacitance indicates the formation of AGEs, which is directly dependent on the concentration of glucose in the solution. The results demonstrate that capacitance measurement could be a reliable tool for measuring the level of glycation, predicting the level of glucose by monitoring the level of glycation, and studying the electrokinetics of glycation. Additionally, the pH of a solution and bacterial growth are important factors that can change the capacitance of the solution [49]. Therefore, we constantly monitored the pH and bacterial contamination in the solution during incubation to ensure that the change in capacitance is solely due to glycation. Furthermore, denatured proteins have different dielectric properties and electrophoretic mobilities than native proteins [50,51]. Therefore, the stable capacitance of BSA and gelatin without glucose throughout the 28 days indicates the stability of proteins in the solution (Figure S3). Cumulatively, the results indicate the potential of the developed device for the evaluation of glycation in clinical settings, and by extension, for in vivo analysis after simple sample processing.

### 3.2. Evaluation of Antiglycation Property by Electrochemical Chip

**Figure 6:**
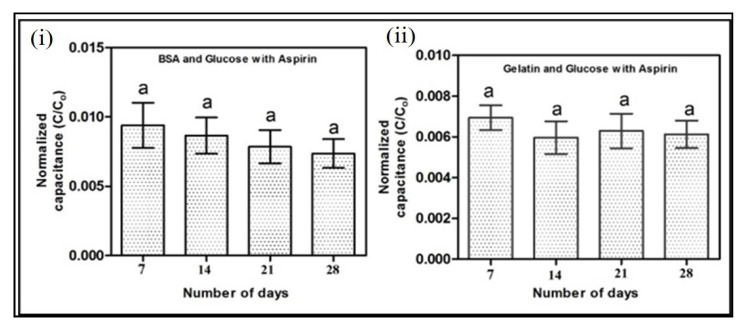
Experimental values of normalized capacitance of (**i**) 40 g/L BSA and 300 mg/dL glucose solution with 40 mmol/L aspirin and (**ii**) 2% gelatin and 300 mg/dL glucose solution with 40 mmol/L aspirin. Incubation time is 28 days for both the samples. *p* < 0.05 was considered statistically significant. Same letters indicate no significant differences between groups. Error bars represent the standard deviation.

The in vitro assessment of antiglycation activity is a primary aspect of antidiabetic drug development. Considering the fast, reproducible, and label-free nature of the electrochemical chip for the evaluation of protein glycation, we further investigated the potential of the electrochemical chip for the assessment of antiglycation activity. No change in the normalized capacitance was observed in 28 days when the BSA and gelatin were incubated with glucose in the presence of aspirin (Figure 6). Previous in vitro studies have suggested that aspirin can inhibit glycation by acetylating reactive lysine residues, binding noncovalently to the proteins, scavenging reactive oxygen species, and chelating the metal ion [37,38,52]. The aforementioned mechanism of aspirin can inhibit the formation of the Schiff base and Amadori products, thereby significantly decreasing the generation of AGEs, MG, and GO. In addition, aspirin can decrease the crosslinking of the generated AGEs, MG, and GO and stabilize the capacitance after 14 days of incubation [38]. Hence, the constant thickness of the electric double layer may be due to the antiglycation nature of the aspirin, resulting in uniform capacitance throughout the 28 days. The developed electrochemical chip can be described as a qualitative that can identify the glycation level and identify the drugs inhibiting glycation. Insulin, another potent antihyperglycemic agent, can reduce the level of glucose in the blood which can lead to reduced glycation of proteins. This chip can also be utilized to measure the efficiency of indirect drugs such as insulin by injecting the drugs in model animals and analyzing their plasma. However, more studies are required before utilizing the plasma samples because, unlike conventional methods such as HPLC and ELISA, the developed microfluidic chip cannot perform absolute quantification of glycated protein.

## 4. Conclusions

In this study, a novel in vitro method was developed using a microelectrode-embedded chip scale device for the evaluation of glycation and antiglycation drugs. We demonstrated that the designed chip can detect the change in capacitance occurring due to the glycation of BSA and gelatin and thus measure the level of protein glycation qualitatively. Additionally, supplementing the glucose and protein solution with aspirin restricted the change in the normalized capacitance over 28 days. Thus, we established that the developed electrochemical chip can be a potential tool for screening antiglycation compounds with only 100 µL of the sample. Because the extracted natural products or synthesized chemicals used as antiglycation agents are usually obtained in minute quantity, the low sample requirement will be useful in preserving the invaluable bioactive compounds. The method is label-free, does not require sample processing steps such as dialysis or column-based separation, and is independent of harmful dyes such as NBT. However, the natural system is far more complex, including the constant inflow of new reducing sugars, lipids, glycoproteins, and free sugar antioxidants. Therefore, this study should not be used for in vitro stored blood. Currently, because of the absence of electrochemical standards for the quantification of AGEs or a universally defined electrochemical AGE values, the quantification and comparative assessment of AGEs between research laboratories is problematic and restricts the usefulness of the chip. Comparative assessment of the electrochemical chip by using conventional methods such as LC-MS, HPLC, or MALDI-TOF is required to establish the quantitative usefulness of electrochemical chip. Furthermore, calibration of free glucose, free protein, AGE, MG, and GO, along with calculating the limit of detection for each component, is needed to understand the utility of the developed chip to study human samples.

## Supplementary Materials

The following are available online at https://www.mdpi.com/1999-4923/12/11/1011/s1, Figure S1. Extent of glycation was measured in the (a) 40 g/L BSA with different concentrations of glucose (0, 150, 300, 450, 600, and 750 mg/dL), and (b) 2% gelatin with different concentrations of glucose (0, 150, 300, 450, 600, and 750 mg/dL). The study was performed after 28 days of incubation, using Nitro Blue tetrazolium assay and reading at 530 nm. Figure S2. LC-MS spectra of (a) carboxymethyl lysine (CML) standard (b) Zero-day BSA (40 g/L BSA and 300 mg/dL after 0 days of incubation) (c) 28-day BSA (40 g/L BSA and 300 mg/dL after 28 days of incubation). Figure S3. Experimental values of capacitance (i) solution of 40 g/L BSA and 40 mmol/L aspirin without glucose and (ii) 2% gelatin and 40 mmol/L aspirin without glucose. Incubation time is 28 days for the both the samples. *p* < 0.05 was considered statistically significant. Same letters indicate no significant differences between groups. Error bars represent the standard deviation.

## Abbreviations

AGEs	advanced glycation end products
BSA	bovine serum albumin
CAD	computer-aided design
CML	N^ε^-(carboxymethyl)lysine
ELISA	enzyme-linked immunosorbent assay
GO	glyoxal
HPLC	high-performance liquid chromatography
LC-MS	liquid chromatography-mass spectrometry
MALDI-TOF	matrix-assisted laser desorption ionization time-of-flight
MG	methylglyoxal
NBT	nitroblue tetrazolium
PDMS	polydimethylsiloxane
