# Supplementary material for: An Electrochemical Chip to Monitor In Vitro Glycation of Proteins and Screening of Antiglycation Potential of Drugs
Source: Pharmaceutics. 2020 Oct 23;12(11):1011. doi: 10.3390/pharmaceutics12111011 (PMC7690698; doi:10.3390/pharmaceutics12111011)
Supplement: Supplementary file 1 [file pharmaceutics-12-01011-s001.pdf]

pharmaceutics

Supplementary Materials: An Electrochemical Chip to Monitor In Vitro Glycation of Protein and Screening of Antiglycation Potential of Drugs

Zeeshan A. Khan and Seungkyung Park

1. Estimation of Glycation by Nitro Blue Tetrazolium (NBT)

Glycation analysis was carried in accordance with the protocol of published elsewhere [21,43,44]. The incubated BSA and gelatin samples were diluted with carbonate buffer (0.1 M, pH 10.3) to achieve a concentration of 5 μM. Both the samples were then incubated at 37 °C for 10 min, followed by the addition of prewarmed NBT (Sigma Aldrich, St. Louis, MO, USA) at a final concentration of 0.03 mM. A blue color develops which was read at 530 nm in a spectrophotometer.

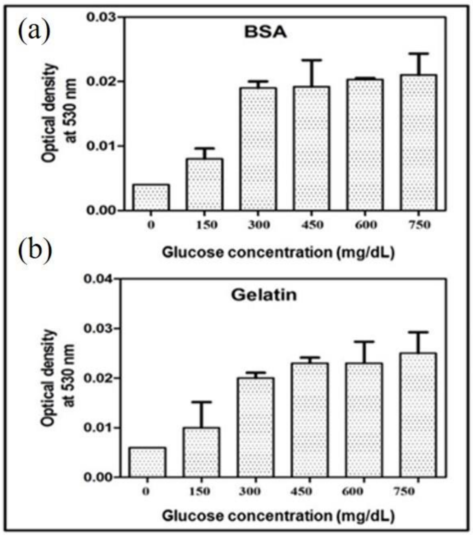

Figure S1. Extent of glycation was measured in the (**a**) 40 g/L BSA with different concentrations of glucose (0, 150, 300, 450, 600, and 750 mg/dL), and (**b**) 2% gelatin with different concentrations of glucose (0, 150, 300, 450, 600, and 750 mg/dL). The study was performed after 28 days of incubation, using Nitro Blue tetrazolium assay and reading at 530 nm.

2. Liquid Chromatography Mass Spectroscopic Analysis (LCMS)

LCMS analysis was performed with zero-day BSA sample (40 g/L BSA and 300 mg/dL after 0 days of incubation) and 28 days-BSA sample (40 g/L BSA and 300 mg/dL after 28 days of incubation) for the determination of AGEs related adduct by using previously published protocol [11]. Carboxymethyl-lysine (CML) was utilized as the AGEs standard. Briefly, the samples were hydrolysed followed by filtration loaded to reverse phase separation assembly attached with the capillary HPLC system equipped with a C 18 analytical column. A solvent A (0.4% acetic acid) and solvent B (0.2% acetonitrile) comprising of 2% formic acid were employed to attain optimum chromatographic conditions. Afterwards, the MS analysis (Micromass Castro Ultima Triple Quadrupole Mass spectrometer, Waters, MA, USA) was done in positive ion mode through the scan range from 0–400 *m*/*z*.

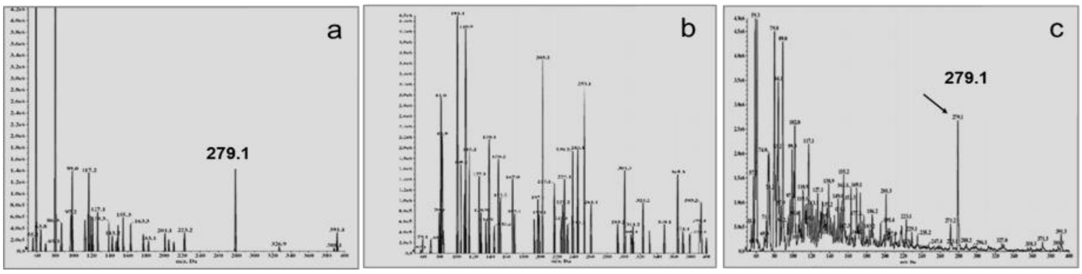

Figure S2. LC-MS spectra of (**a**) carboxymethyl lysine (CML) standard (**b**) Zero-day BSA (40 g/L BSA and 300 mg/dL after 0 days of incubation) (**c**) 28-day BSA (40 g/L BSA and 300 mg/dL after 28 days of incubation).

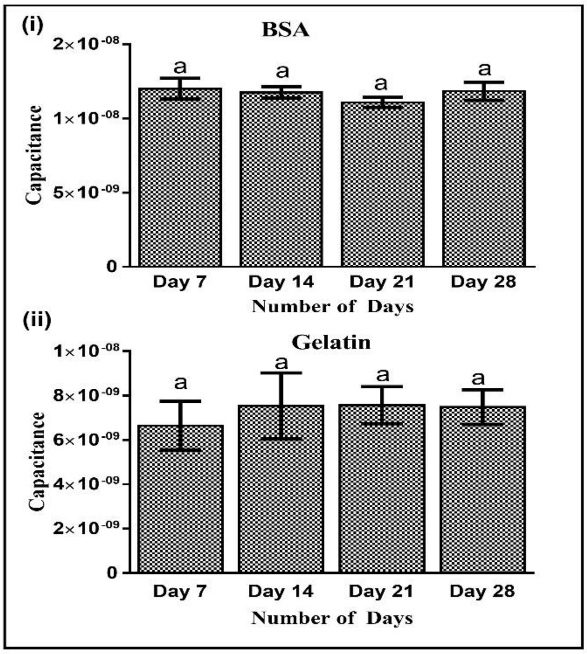

Figure S3. Experimental values of capacitance (**i**) solution of 40 g/L BSA and 40 mmol/L aspirin without glucose and (**ii**) 2% gelatin and 40 mmol/L aspirin without glucose. Incubation time is 28 days for the both the samples. $p < 0.05$ was considered statistically significant. Same letters indicate no significant differences between groups. Error bars represent the standard deviation.
